# Supplementary material for: Effects of Spermine Synthase Deficiency in Mesenchymal Stromal Cells Are Rescued by Upstream Inhibition of Ornithine Decarboxylase
Source: Int J Mol Sci. 2024 Feb 20;25(5):2463. doi: 10.3390/ijms25052463 (PMC10931026; doi:10.3390/ijms25052463)
Supplement: Supplementary file 1 [file ijms-25-02463-s001.zip › ijms-2855956-supplementary.pdf]

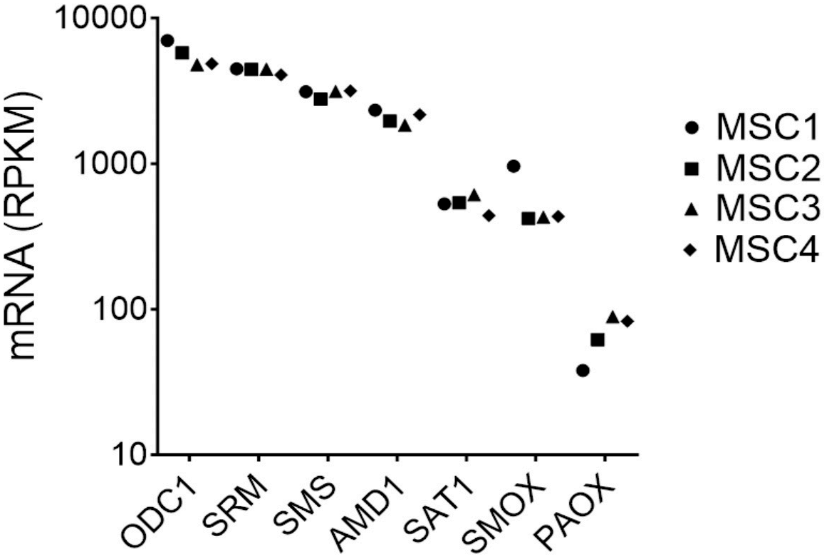

Supplementary Figure S1. mRNA levels of polyamine-associated enzymes, as measured using RNAseq. Gene expression in untreated MSCs derived from four different donors is shown. Notice that SAT1 and SMOX are expressed at approximately 10-fold lower levels than ODC1, SRM, and SMS, while PAOX mRNA is about 10-fold lower than in SAT1 and SMOX. For original RNAseq-associated methods, see Awan et al. 2018 [41].

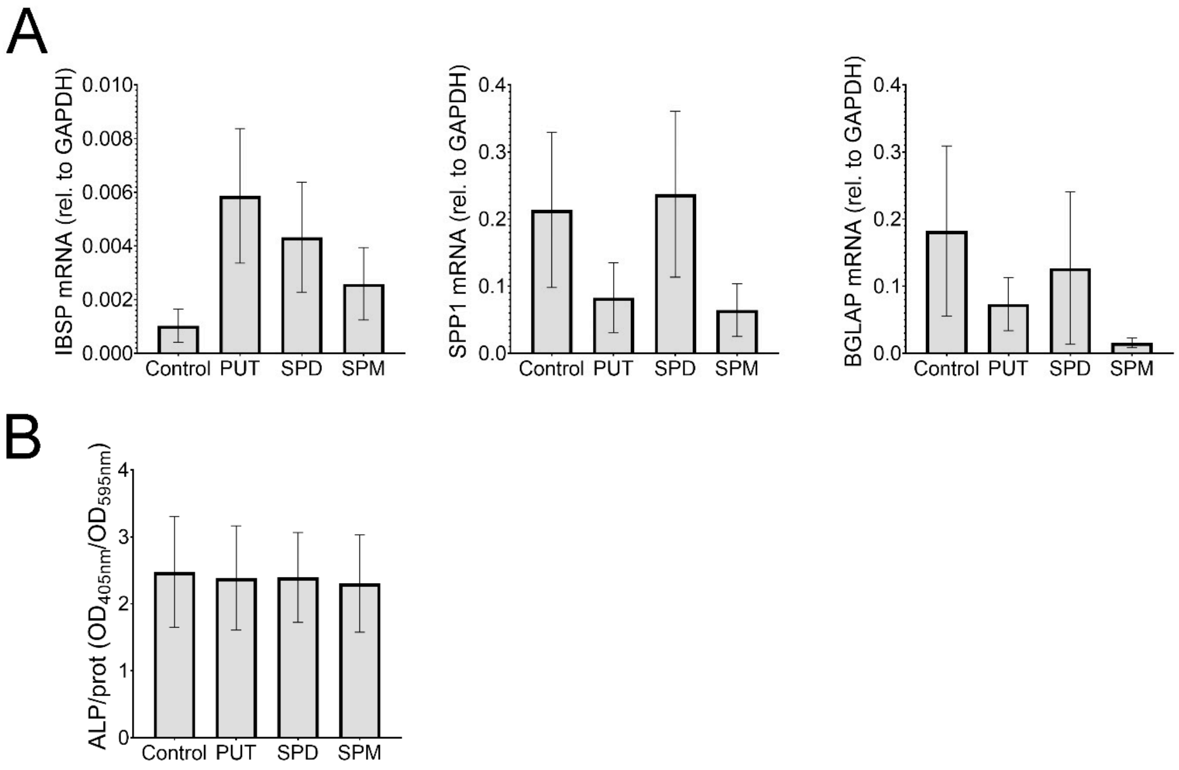

Supplementary Figure S2. Expression of osteogenic genes and alkaline phosphatase (ALP) activity in MSCs supplemented with polyamines. (**A**) Expression of osteogenic markers measured after 14 days in osteogenic media, supplemented with aminoguanidine and polyamines (20 μM; n = 4). (**B**) ALP

activity was measured after 10 days in osteogenic media supplemented as above (n = 7).

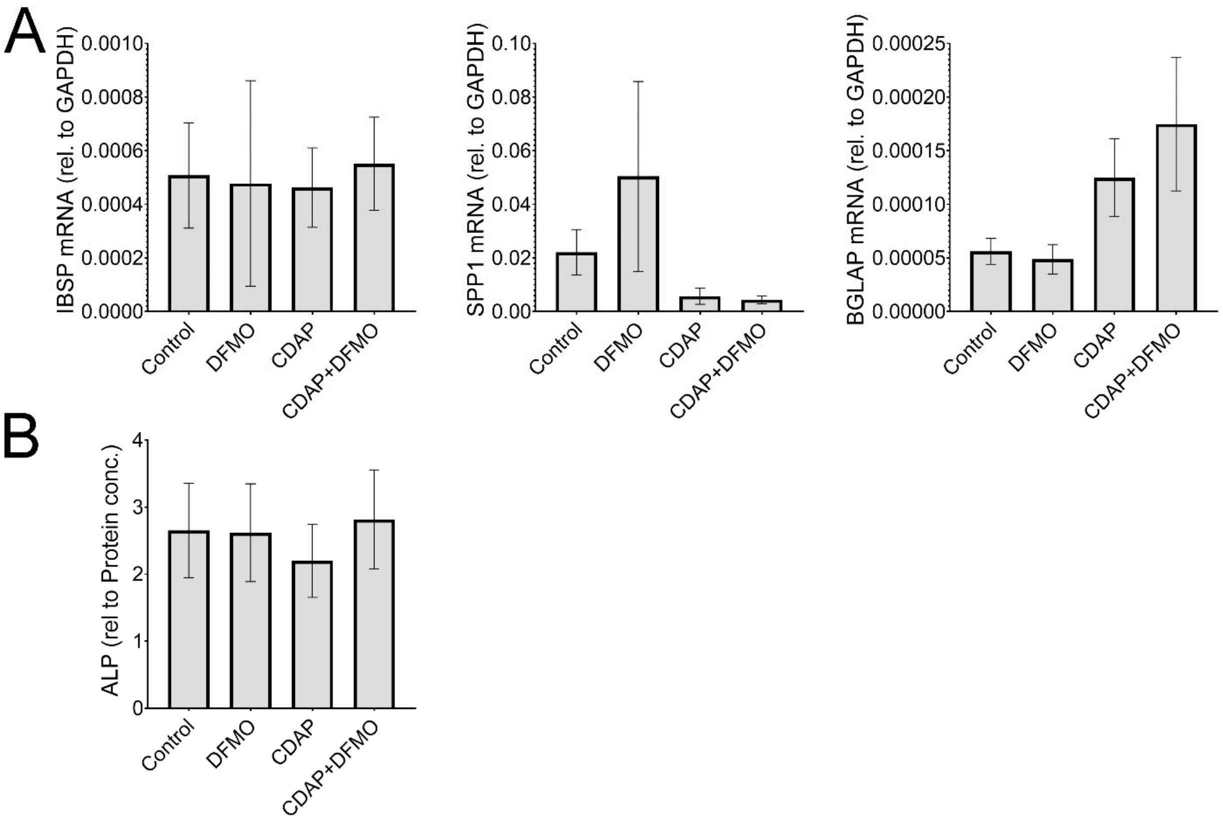

Supplementary Figure S3. Expression of osteogenic genes and alkaline phosphatase (ALP) activity in MSCs supplemented with inhibitors. (**A**) Expression of osteogenic markers measured after 14 days in osteogenic media, supplemented with inhibitors (CDAP at 200 µM and DFMO at 10 µM; n = 4) (**B**) ALP activity was measured after 10 days in osteogenic media supplemented as above (n = 6).

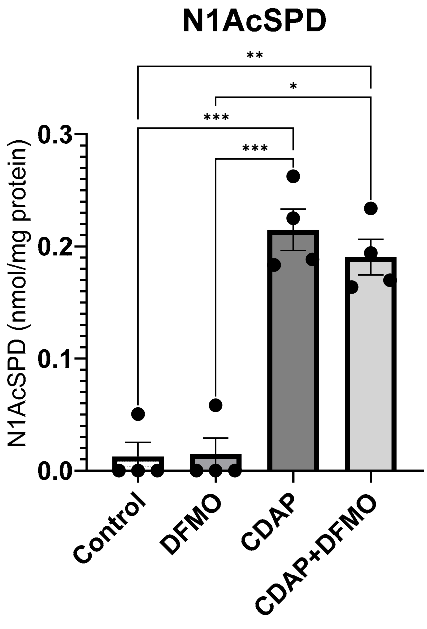

Supplementary Figure S4. CDAP increases N1AcSPD levels, which are only slightly reduced with DFMO. MSCs were cultured for 48 h with either

DFMO (20 μM) or CDAP (100 μM) and processed for either polyamine measurements. Acetylated spermidine (N1AcSPD) was measured using HPLC (n = 4). Acetylated spermine was below detection levels (not shown). * $p < 0.05$; ** $p < 0.005$; *** $p < 0.0005$ as calculated by 1-way ANOVA and post hoc Tukey's test.

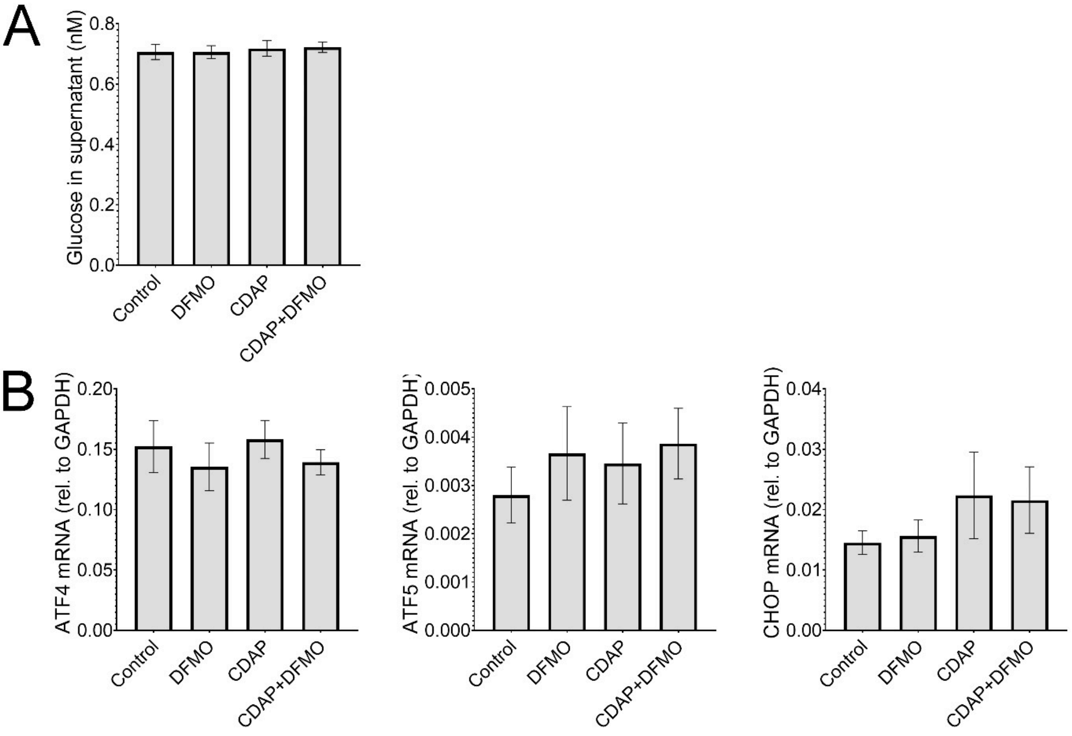

Supplementary Figure S5. Glucose consumption and expression of mitochondrial stress response genes in response to CDAP and DFMO. (**A**) Glucose concentration in the supernatant of MSCs cultured for 48 h with either CDAP (200 μM), DFMO (10 μM), or both (n = 5). (**B**) Expression of mitochondrial stress response genes ATF4, ATF5, and CHOP in MSCs cultured for 24 h with either CDAP (200 μM), DFMO (10 μM), or both (n = 11).
